# Evaluation of OPTIMISE (Online Programme to Tackle Individual’s Meat Intake Through Self-regulation): Cohort Study

**DOI:** 10.2196/37389

**Published:** 2022-12-12

**Authors:** Cristina Stewart, Carmen Piernas, Kerstin Frie, Brian Cook, Susan A Jebb

**Affiliations:** 1 Nuffield Department of Primary Care Health Sciences University of Oxford Oxford United Kingdom

**Keywords:** self-regulation, self-monitoring, goal setting, meat intake, meat reduction, multi-component intervention, health, nutrition, diet

## Abstract

**Background:**

There is an urgent need to reduce society’s meat consumption to help mitigate climate change and reduce noncommunicable diseases.

**Objective:**

This study aimed to investigate changes in meat intake after participation in an online, multicomponent, self-regulation intervention.

**Methods:**

We conducted a pre-post observational study among adult meat eaters in the United Kingdom who signed up to a website offering support based on self-regulation theory to reduce meat consumption. The program lasted 9 weeks (including a 1-week baseline phase, a 4-week active intervention phase, and a 4-week maintenance phase), comprising self-monitoring, goal setting, action planning, and health and environmental feedback. Meat intake was estimated during weeks 1, 5, and 9 using a 7-day meat frequency questionnaire. We analyzed the change in mean daily meat intake from baseline to week 5 and week 9 among those reporting data using a hierarchical linear mixed model. We assessed changes in attitudes toward meat consumption by questionnaire and considered the acceptability and feasibility of the intervention.

**Results:**

The baseline cohort consisted of 289 participants, of whom 77 were analyzed at week 5 (26.6% of the baseline sample) and 55 at week 9 (71.4% of the week 5 sample). We observed large reductions in meat intake at 5 and 9 weeks: –57 (95% CI –70 to –43) g/day (*P*<.001) and –49 (95% CI –64 to –34) g/day (*P*<.001), respectively. Participants’ meat-free self-efficacy increased, meat-eating identities moved toward reduced-meat and non–meat-eating identities, and perceptions of meat consumption as the social norm reduced. Participants who completed the study reported high engagement and satisfaction with the intervention.

**Conclusions:**

Among people motivated to engage, this online self-regulation program may lead to large reductions in meat intake for more than 2 months, with promising signs of a change in meat-eating identity toward more plant-based diets. This digital behavior change intervention could be offered to complement population-level interventions to support reduction of meat consumption.

## Introduction

Population-level changes in meat consumption are needed to help mitigate climate change and reduce noncommunicable diseases. The livestock sector is a leading contributor to environmental degradation [1], while a high intake of meat, particularly red and processed meat, has been linked to type 2 diabetes, cardiovascular disease, and some forms of cancer [2]. There is a growing interest in reduced-meat diets, primarily for health reasons, but also because of concerns regarding animal welfare and the environment [3]. According to UK public attitude surveys, 65% of people surveyed in 2020 were willing to consider eating less meat [3], up from 35% in 2014 [4]. Meat substitutes are also rising in popularity; a trend analysis of the UK National Diet and Nutrition Survey (NDNS) found that their consumption has almost doubled in the last decade [5], and market research data suggests the number of British people eating these products has increased from 50% in 2017 to 65% in 2019 [6]. However, meat consumption in the United Kingdom is decreasing only slowly (–17 g/capita/day; –17% in the last decade) [7], suggesting people need more support to enact their intentions to reduce meat in their diet and close the intention-behavior gap.

Individual-level interventions (targeting our conscious and reflective decision-making processes) can complement interventions at a population level (targeting automatic, nonconscious processes) [8], but need to be delivered at scale [9-11]. Using digital technology (eg, mobile apps, interactive websites, and text messaging) is a promising approach to providing scalable, cost-effective interventions [12], and evidence suggests this approach can help promote a range of healthy behaviors [12-15]. Previous research has noted that these interventions need to be thoroughly grounded in behavior change theory [16].

We recently developed an online multicomponent intervention, OPTIMISE (Online Programme to Tackle Individual’s Meat Intake Through Self-regulation), based on self-regulation theory to support individuals in reducing their meat consumption. The intervention guides individuals through a self-regulation process of self-monitoring, goal setting, learning about the health and environmental impact of their meat intake, action planning, and regular reflection. We tested its effectiveness in a randomized controlled trial (RCT) [17] among adults who ate meat very regularly (≥5 times per week), and found it led to significant reductions in meat intake: a 40 g/day greater reduction, relative to the control group, at 5 weeks. Identifying effective and potentially scalable interventions that can support people’s efforts to enact their intentions to eat less meat is imperative to improve both planetary and human health.

This population-based cohort study builds upon our previous RCT and aims to investigate whether this online self-regulation intervention is effective in helping the general population in the United Kingdom who eat at least some meat to reduce their meat intake. A secondary aim was to investigate the adherence to and acceptability of the intervention.

## Methods

### Study Design and Setting

We conducted a cohort study among UK adults using OPTIMISE, an online program to support meat reduction based on self-regulation theory. All aspects of the study were delivered remotely through a website developed specifically for the intervention, through which all data collection took place between May 28, 2021, and December 13, 2021.

### Ethics Approval

This study was granted ethical approval by the Central University Research Ethics Committee of the University of Oxford (R71430/RE003).

### Participant Recruitment

We made the website publicly available and signposted it to people over 6 months through public engagement events across the United Kingdom via our research team’s website and social media presence, as well as online newsletters and volunteer databases (eg, Research for the Future) [18]. Everyone who accessed the OPTIMISE website was offered the opportunity to sign up as a study participant or use the program independently on their own. Recruitment closed 1 month after our last public engagement event.

People interested in taking part in the research completed a screening questionnaire to assess eligibility (participants were aged 18 years or older, were resident in the United Kingdom, were meat eaters, and wanted to reduce their meat intake), and they provided consent for participation in the study before registering with the OPTIMISE website using their email address. Participants who completed the program were entered into a raffle to win a £100 (US $122.53) digital gift card (1 gift card was available for every 50 participants).

### Study Procedures

**Figure 1:**
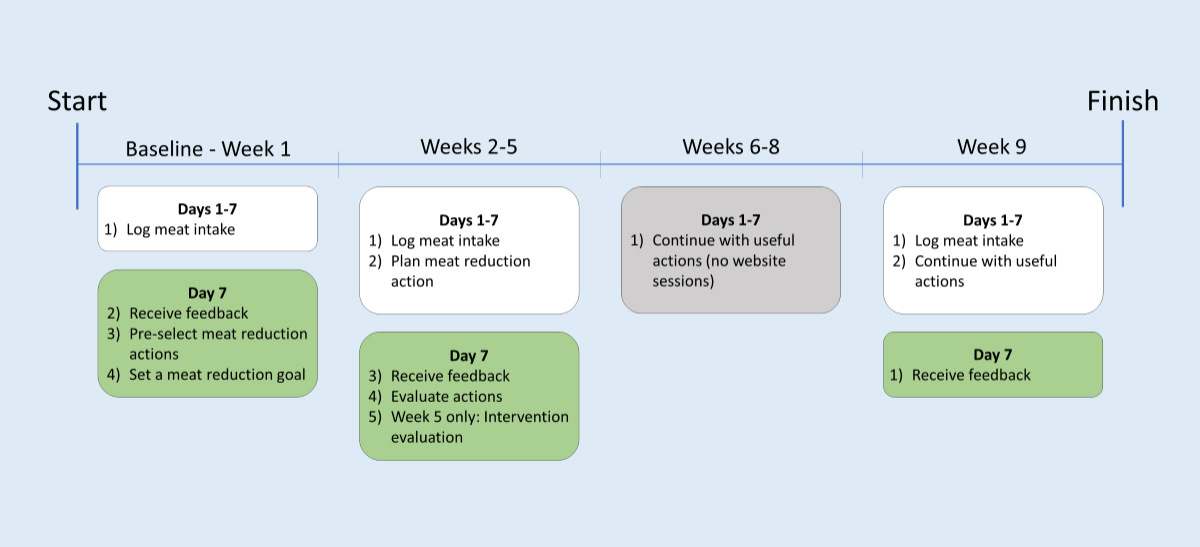
Procedure of the OPTIMISE (Online Programme to Tackle Individual’s Meat Intake Through Self-regulation) study.

The study lasted 9 weeks (including a baseline week of self-monitoring meat consumption, a 4-week active intervention phase, and a 4-week maintenance phase; Figure 1), with 3 data collection weeks: baseline (week 1), first follow-up (week 5) and second follow-up (week 9). After registering with the OPTIMISE website, participants were presented with information regarding the health and environmental benefits of eating less meat (Multimedia Appendix 1). Participants then completed a baseline questionnaire that asked about their demographic characteristics, dietary restrictions, and attitudes toward meat consumption. The attitudes assessed were meat-eating identity, meat-free self-efficacy (adapted from Lacroix and Gifford’s self-efficacy scale [19]), motivation to reduce meat consumption, perception of meat consumption as the social norm (consisting of the 4 *N*’s—the belief that eating meat is “natural, normal, necessary, and nice” [20]), and social support for meat reduction. Full details are provided in Multimedia Appendix 2. Participants repeated the attitude questionnaire at both follow-ups (at weeks 5 and 9). Meat consumption was measured daily during the 3 data collection weeks using a specific meat frequency questionnaire [21]. This questionnaire asked participants to report the number of servings of individual meat and seafood items they consumed in the previous 24 hours. Serving sizes were based on underlying portion size data from the UK Food Standards Agency combined with estimates of meat content from composite dishes from the UK NDNS [22,23].

### The Intervention

The full intervention has been described in detail previously [17]. In short, on the last day of the baseline week, participants received feedback on the health and environmental impacts of their total meat consumption and red meat consumption. They were then presented with a list of 26 meat consumption reduction actions across 6 categories that they could preselect for the upcoming weeks: (1) “preparing to change”; (2) “try swapping out meat for veg”; (3) “try something new”; (4) “cut out specific animal products”; (5) “limit intake of animal products”; and (6) “get family and friends involved” (Multimedia Appendix 3). The participants were also prompted to set themselves a goal to reduce their meat consumption. Every day throughout the active intervention phase (ie, weeks 2-5), participants self-monitored their meat consumption and planned a meat reduction action. Each subsequent morning they were asked if they had managed to perform their action on the previous day; if they had not, they were asked to reflect on what they could do differently next time. At the end of each week of the active intervention phase, the participants received feedback on how their meat consumption compared to baseline (Multimedia Appendix 4), and they were asked to reflect on how useful they found the actions they had chosen that week. At the first follow-up (week 5), participants completed an intervention evaluation questionnaire. During the 4-week maintenance phase (ie, weeks 6-9), the participants were asked to continue performing the actions they found useful during the active intervention phase offline, with no web sessions to complete.

### Outcome Measures

The main outcome measure was the change in mean daily meat consumption from baseline to week 5, measured by the daily meat frequency questionnaires [21]. We also assessed the change in (1) total mean daily meat consumption from baseline to week 9, (2) total mean daily meat consumption from week 5 to week 9, and (3) mean daily consumption of meat subtypes comprising red meat and processed meat from baseline to weeks 5 and 9.

We also explored the predictors of change in meat intake and change in attitudes toward meat consumption from baseline to weeks 5 and 9. We assessed adherence to the intervention as the proportion of the 42 sessions participants completed and the acceptability of the intervention based on responses to the intervention evaluation questionnaire.

### Statistical Analysis

All statistical analyses were conducted in Stata/IC (version 14.1). *P*<.05 was set to denote statistical significance. We published a statistical analysis plan on the Open Science Framework preceding the analyses on October 18, 2021 [24].

For each participant and time point (baseline, week 5, and week 9), we calculated mean total daily intakes of all meat and meat subtypes (ie, red meat and processed meat). The main analysis used a hierarchical linear mixed model with fixed effects for “time point” and random effects for “participant” to investigate whether meat consumption at weeks 5 and 9 differed significantly from baseline. As prespecified in our statistical analysis plan, days in which reported meat intake exceeded 1.5 kg were excluded, as we deemed this implausible. We identified no confounding variables through univariable regressions and so the model was unadjusted.

To analyze the predictors of change in mean daily meat consumption from baseline to week 5, we used a multivariable linear regression model with change in meat consumption as the dependent variable and possible predictors included in one single model. The predictors were age, gender, ethnic group, highest educational qualification, household size, annual household income, the response to “currently trying to lose weight” (yes/no), dietary restrictions, baseline meat consumption, baseline attitudes toward meat (ie, meat-eating identity, including non–meat eater, reduced-meat eater, and meat eater; mean meat-free self-efficacy; meat reduction motivation; mean meat consumption social norms; and meat reduction social support), tertiles of engagement (based on the percentage of sessions participants completed throughout the active intervention phase), and the number of action categories tried at least once.

We used hierarchical linear mixed models to investigate changes in attitudes toward meat (ie, meat-free self-efficacy, meat reduction motivation, meat consumption social norms, and meat reduction social support), between baseline and weeks 5 and 9. Due to multicollinearity between tertiles of engagement and meat-eating identity changes, we used the chi-square goodness-of-fit test to explore the proportions of each meat-eating identity at both follow-ups compared to baseline. Written feedback collected from participants as part of the intervention evaluation questionnaire was analyzed qualitatively using inductive thematic analysis in NVivo 12 (QSR International) [25].

Sensitivity analyses were performed using 2-tailed independent *t* tests (for normal continuous data), Mann-Whitney *U* tests (for skewed continuous data), and chi-square tests (for categorical data) to explore baseline differences in participants who did not provide any outcome data (ie, who did not complete any sessions in week 5) and those who did.

### Exploratory Analyses

To explore barriers to adherence to participants’ chosen meat reduction actions, we analyzed the free-text responses to the daily action completion question when participants indicated they had not managed to perform their action using inductive thematic analysis [25].

## Results

### Participants

A total of 566 individuals signed up to the study website, 59 of whom requested their account (and subsequently all their data) be deleted before the end of the study. We were unable to establish which of these 59 individuals were study participants and which were independent users. Of the remaining 507 individuals for whom we had data, 120 registered as independent users and 387 registered as study participants.

Of the study participants, 82 did not complete any baseline sessions, 3 did not complete the baseline demographics questionnaire, and 7 reported no meat consumption during the baseline week. Six participants were excluded as they self-reported eating >1.5 kg of meat per day in every meat frequency questionnaire they completed. The total baseline cohort, therefore, consisted of 289 of the 387 registered participants (74.7%). Participants were aged 18 to 84 years (mean 46.8, SD 13.8 years), 72.3% (209/289) were female, and 57.1% (165/289) were White British (Table 1). Reported total meat consumption at baseline was 146 (SD 162) g/day (Table 2).

**Figure 2:**
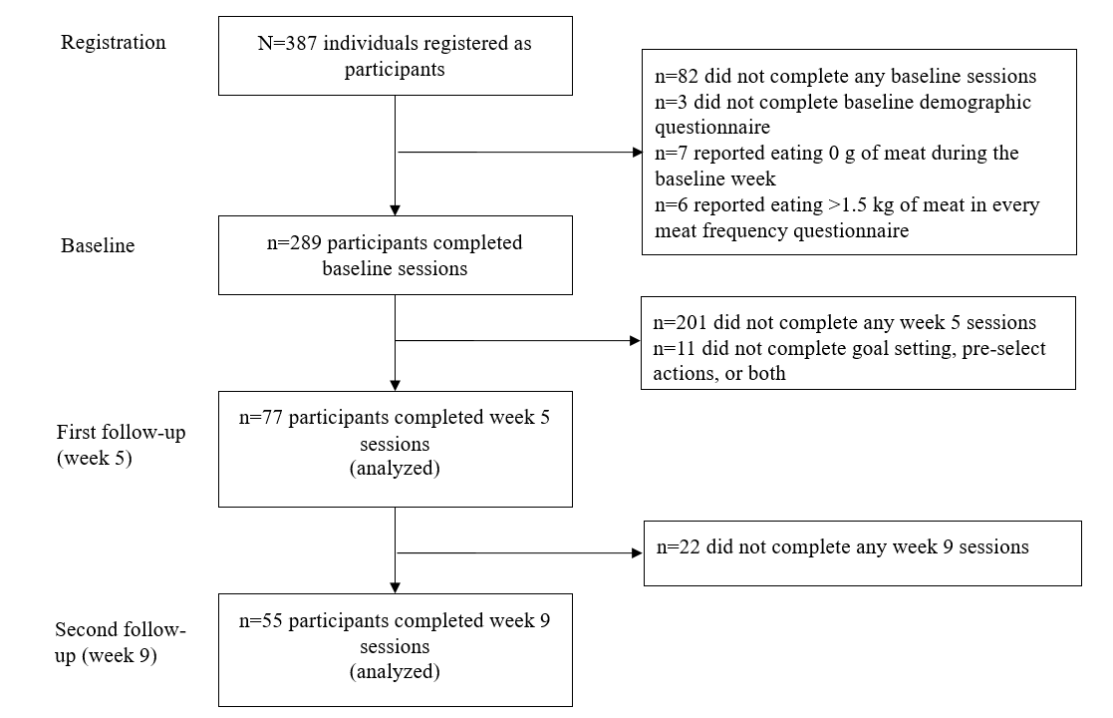
Flow chart of participants.

Eleven participants did not complete their goal setting, preselect their actions, or both, and a further 201 participants did not complete any sessions in week 5, leaving 77 participants in our first follow-up sample (week 5; 26.6% of the baseline sample of 289 participants). Of these participants, 22 did not complete any sessions in week 9, leaving 55 participants in our second follow-up sample (week 9; 71.4% of the first follow-up sample of 77 participants). Figure 2 depicts a flow diagram of participants throughout the study.

In the baseline cohort, the most important motivating factor to reduce meat intake on a scale from 1 (not at all important) to 10 (extremely important) was to help the environment (mean score 8.6, SD 1.5), followed by health benefits (mean score 7.9, SD 1.8) and animal welfare concerns (mean score 7.6, SD 2.3). The mean meat-consumption reduction goal shows participants on average challenged themselves to reduce their meat consumption by nearly a quarter (–23%, SD 13%; range 5%-90%).

Participants who dropped out before week 5 were more likely to be trying to lose weight (*P*=.03) and were less motivated to reduce their meat consumption at baseline (*P*=.02) compared to those who completed week 5 sessions. No other baseline measurements differed significantly between these groups (Multimedia Appendix 5).

**Table 1:** Baseline characteristics (N=289).

Characteristics		Values	
Age^a^ (years), mean (SD)		46.8 (13.8)	
**Gender, n (%)**			
	Female		209 (72.3)
	Male		78 (27)
	Other/prefer not to say		2 (0.7)
**Ethnicity, n (%)**			
	White British		165 (57.1)
	White other		84 (29.1)
	Asian or Asian British		17 (5.9)
	Black or Black British		4 (1.4)
	Mixed/other		18 (6.2)
	Prefer not to say		1 (0.4)
**Highest educational qualification, n (%)**			
	University degree, NVQ^b^ level 4-5 or equivalent, and above		242 (83.7)
	Other post–high school qualifications		15 (5.2)
	A-levels^c^, NVQ level 2-3 or equivalent		21 (7.3)
	Apprenticeship		1 (0.4)
	GCSE^d^, NVQ level 1, or equivalent		2 (0.7)
	Other vocational, work-related qualifications		3 (1)
	No formal qualifications		1 (0.4)
	Prefer not to say		4 (1.4)
**Household size, n (%)**			
	1 person		55 (19)
	2 people		115 (39.8)
	3 people		57 (19.7)
	4 people		48 (16.6)
	5 people		10 (3.5)
	≥6 people		4 (1.4)
**Annual household income, n (%)**			
	<£15,000 (US $18,418)		10 (3.5)
	£15,000-£24,999 (US $18,418-$30,695)		24 (8.3)
	£25,000-£39,999 (US $30,695-$49,113)		45 (15.6)
	£40,000-£75,000 (US $49,113-$92,090)		99 (34.3)
	>£75,000 (>US $92,090)		90 (31.1)
	Prefer not to say		21 (7.3)
**Currently trying to lose weight, n (%)**			
	Yes		198 (68.5)
	No		91 (31.5)
**Dietary restrictions^e^, n (%)**			
	Dairy-free		14 (4.9)
	Gluten-free		19 (6.6)
	Fish and shellfish allergy		3 (1)
	None		259 (89.6)
**How participants heard of the program, n (%)**			
	Public engagement events		4 (1.4)
	Research team’s website/social media		6 (2.1)
	Friends or family members		17 (5.9)
	Social media		48 (16.6)
	Radio or newspaper		181 (62.6)
	Volunteer databases		9 (3.1)
	Other		24 (8.3)

^a^Age ranged from 18 to 84 years.

^b^NVQ: National Vocational Qualification.

^c^Advanced level (A-level) qualifications are subject-based qualifications for students aged 16 or older.

^d^GCSE: General Certificate of Secondary Education.

^e^Participants could select multiple answers to this question.

**Table 2:** Meat consumption and attitudes at baseline and both follow-ups. Estimates are from mixed effects models with fixed effects for “time point” and random effects for “participant.” The models were unadjusted, as we identified no potential confounders in univariate analyses. Data on meat-eating identity are shown in Multimedia Appendix 6. Baseline N=289; meat consumption n=77 and n=55 participants at first and second follow-ups, respectively; attitudinal measures, n= 55 and n=41 participants at first and second follow-ups, respectively.

		Baseline, mean (SD)	First follow-up (week 5)				Second follow-up (week 9)		
			Mean (SD)	Mean difference (95% CI)	*P* value	Mean (SD)		Mean difference (95% CI)	*P* value
**Meat consumption (g/day)**									
	Total meat	146 (162)	61 (50)	–57 (–70 to –43)	<.001	68 (51)		–49 (–64 to –34)	<.001
	Red meat	53 (65)	27 (33)	–22 (–32 to –12)	<.001	28 (31)		–21 (–32 to –10)	<.001
	Processed meat	40 (80)	17 (23)	–13 (–19 to –8)	<.001	18 (22)		–12 (–18 to –5)	<.001
	Red and processed meat	92 (121)	44 (52)	–35 (–49 to –22)	<.001	46 (48)		–33 (–48 to –18)	<.001
**Attitudinal measures**									
	Meat-free self-efficacy score^a^	3.2 (1.2)	2.8 (1.4)	–0.3 (–0.6 to 0.0)	.09	2.4 (1.1)		–0.8 (–1.2 to –0.5)	<.001
	Meat reduction motivation score^b^	7.5 (1.6)	8.1 (1.6)	0.2 (–0.3 to 0.8)	.45	7.7 (2.1)		–0.1 (–0.7 to 0.5)	.72
	Meat consumption social norms score^c^	4.4 (1.0)	4.2 (1.2)	–0.2 (–0.4 to 0.0)	.13	4.0 (1.1)		–0.4 (–0.6 to –0.1)	.001
	Meat reduction social support score^d^	6.6 (2.5)	7.0 (2.6)	–0.1 (–0.8 to 0.5)	.66	6.3 (2.5)		–0.8 (–1.5 to –0.1)	.02

^a^Mean score of 3 self-efficacy questions (“I lack the cooking skills to prepare meat-free meals,” “I don’t know what to eat instead of meat,” and “I don’t have enough willpower to not eat meat”), measured on a scale from 1 (strongly disagree) to 7 (strongly agree).

^b^Participants were asked to respond to the question “How motivated are you to reduce your meat intake beyond the context of this programme?” on a scale from 1 (not at all motivated) to 10 (extremely motivated).

^c^Mean score of responses to 4 social norm questions using the 4 *N*’s scale (the belief that eating meat is “natural, normal, necessary, and nice”) on a scale from 1 (strongly disagree) to 7 (strongly agree).

^d^Participants were asked, “How willing are the people you share your meals with to reduce their meat consumption?” on a scale from 1 (not open at all) to 10 (very open to it).

### Changes in Meat Consumption

Total mean consumption of meat decreased from baseline to week 5 by –57 (95% CI –70 to –43) g/day (*P*<.001) and from baseline to week 9 by –49 (95% CI –64 to –34) g/day (*P*<.001). This included reductions in consumption of red meat and processed meat of –35 (95% CI –49 to –22) g/day (*P*<.001) and –33 (95% CI –48 to –18) g/day (*P*<.001) at weeks 5 and 9, respectively (Table 2). The reduction in total meat consumption from week 5 to week 9 was –8 (95% CI –7 to –23 g/day), but this was not a significant difference (*P*=.31).

### Predictors of Change

Higher baseline meat consumption was associated with a greater reduction in meat intake at week 5, with every 1 g of greater baseline intake predicting a 0.9 g/day greater reduction (95% CI –1.1 to –0.7; *P*<.001). Choosing meat reduction actions from only one category was associated with an increase in meat consumption from baseline to week 5 of 104 (95% CI 10 to 198) g/day (*P*=.03). For participants choosing actions from more than one category, there was no association between the number of action categories chosen and meat intake reduction. No demographic characteristics or baseline attitudes toward meat significantly predicted change in meat intake, nor did tertiles of intervention engagement (Multimedia Appendix 7).

### Changes in Attitudes Toward Meat Consumption

There was a significant change in reported meat-eating identities toward reduced-meat and non–meat-eating identities from baseline to both follow-ups (*P*=.005 at week 5 and *P*=.002 at week 9). Forty-four percent (23/52) and 43% (17/40) of participants described themselves as meat eaters at weeks 5 and 9, down 25 and 30 absolute percentage points from baseline, respectively (Multimedia Appendix 6). There was no change in any other attitudinal measures from baseline to week 5. At week 9, there was an increase in mean meat-free self-efficacy score (–0.8, 95% CI –1.2 to –0.5; *P*<.001), a decrease in the score for perception of meat consumption as the social norm (–0.4, 95% CI –0.6 to –0.1; *P*=.001), and a decrease in the score for perceived social support for meat reduction (–0.8, 95% CI –1.5 to –0.1; *P*=.02). There was no change in participants’ motivation to reduce meat intake at either week 5 or 9 (Table 2).

### Self-reported Barriers

The most commonly reported barriers for not performing meat reduction actions were (1) other people (most frequently friends and family), (2) being too busy and not having enough time, (3) eating out and the lack of meat-free options available or the temptation to opt for a meat dish, and (4) eating meat leftovers and wanting to avoid food waste. Representative quotes are as follows:

I was not cooking yesterday, and when you’re a guest I think it’s polite to eat what’s been served.

I was very exhausted today and didn’t have the energy to make two dishes.

I ate leftover food, my partner had cooked more meat than the children wanted or needed.

### Acceptability and Feasibility

More than 7 out of 10 participants dropped out before week 5 (73.4%, 212/289), but thereafter, 71% (55/77) completed the study. Of the participants who completed week 5 and week 9, 78% (60/77) and 98% (54/55) completed at least 80% (34/42) of the sessions, respectively. Fifty-five participants (71%, 55/77) completed the intervention evaluation questionnaire at week 5, rating the usefulness of the intervention components and additional resources on a scale from 1 (not useful) to 10 (very useful). Mean scores ranged from 7.2 (SD 2.7) to 9 (SD 1.7) (Table 3). Participants rated the daily meat consumption tracking to be the most useful component of the intervention (mean score 8.7, SD 1.6) and the action diary as the most useful additional resource (mean score 9.0, SD 1.7). Forty-one participants provided additional feedback as responses to the free text question; they were largely positive about their experience of the program. Responses included the following:

In general I’ve found the study interesting and important. It has been effective to chart and reflect on my meat consumption, plan for change and see my evidence of change progressively.

It has helped me to confirm what my personal stumbling blocks are.

To me, tracking the meat consumption and planning activities was the best way to help me out eating less meat, because I’m a naturally planned person.

While some participants said the daily action planning was helpful, others said they would have preferred weekly actions to make planning meals in advance for the week easier. Some participants said they would have liked both social and competitive elements, allowing them to share their progress with others and compare their intake with other users or the UK average, or both.

**Table 3:** Intervention evaluation questionnaire results. Participants were asked how useful they found the items on a scale of 1 (not useful) to 10 (very useful). The additional resources were optional and only evaluated by those who reported using them throughout the study.

Questionnaire items		Mean score (SD)	Respondents, n
**Intervention components**			
	Tracking your meat consumption on a daily basis	8.7 (1.6)	55
	Feedback on the environmental and health impact of your meat consumption	7.6 (2.4)	55
	Planning an action on a daily basis to reduce your meat consumption	7.2 (2.7)	55
**Additional resources**			
	Weekly action evaluation	7.6 (2.4)	55
	Downloadable action diary	9.0 (1.7)	3
	Downloadable action overview	8.0 (2.0)	3
	Links to other resources	8.3 (1.5)	10
	Ability to review your journey	8.2 (1.9)	22

## Discussion

### Principal Results

We observed significant reductions in meat consumption when UK adult meat eaters engaged with a bespoke meat-consumption reduction website and were guided through a process of self-regulation. Participants reported marked changes in meat-eating identity toward reduced-meat and non–meat-eating identities, their meat-free self-efficacy increased, and their perception of meat consumption as the social norm decreased. There was a high dropout rate from registration to first follow-up, but the quarter of participants who provided outcome data had high engagement with the intervention and rated it highly, particularly the self-monitoring aspect.

### Strengths and Limitations

Strengths of this study were that baseline meat intake was similar to that of the general UK population [7] and that we collected detailed estimates of the quantity and type of meat consumed using a specific meat frequency questionnaire [21]. We recruited participants from the general population through public engagement events, social media, and broadcast media, and our results likely reflect the characteristics of people who were attracted to this type of digital self-help support for dietary change. The OPTIMISE program was free to use, easy to sign up to, and easy to try out. To try to mimic “real-world” usage and minimize researcher bias, the participants had no direct contact with the researcher, and all aspects of the intervention were delivered remotely through our study website. Many people who initially signed up as participants did not complete any follow-up assessments, suggesting that those included in the analysis represent a particularly motivated group of people. The high dropout rate was not surprising, as previous research has noted that a high level of attrition poses a significant challenge for digital interventions [26,27], including web-based trials [28,29]. A recent systematic review of app-based interventions for chronic disease found dropout rates were high—up to 87%—with higher rates seen in observational studies than RCTs [26]. Moreover, an observational study testing a healthy-eating app found less than 3% of users were classed as “active” [27], with the majority of participants downloading the app and using it only once. As with other real-world evaluations, another limitation is that we had no randomly assigned control group and cannot infer a causal link between the website and the reduction in meat intake. The reduction in meat intake was maintained 4 weeks beyond the active intervention, and there were associated changes in meat-eating identity, factors that have been shown to be predictive of behavioral intentions [30]. Nevertheless, a longer follow-up period is needed to assess changes in habitual dietary behaviors.

### Comparison With Prior Work

The absolute reduction in meat intake reported here was both large and remarkably similar to the reduction observed in the intervention group in our previous RCT (–58% vs –57% at week 5 and –53% vs –52% at week 9 in the current study and the RCT, respectively) [17]. For context, average meat intake in the United Kingdom has decreased by only 1.7% per year, on average, over the 10 years after 2008-2009 [7]. However, in both studies, we specifically recruited people seeking to reduce their meat intake, and our findings should be interpreted accordingly. We cannot infer causality or precisely identify the active components of the online program, but participants rated self-monitoring as the most useful component. In our RCT, we also observed significant reductions in meat intake in the control group, who were not offered goal setting, action planning, or feedback components but did self-monitor as part of the outcome assessments. It is plausible that the observed reductions in meat consumption are largely a result of self-monitoring. Indeed, previous research has found self-monitoring to be effective in helping individuals to reduce their meat consumption [31,32] and in promoting other positive lifestyle and dietary behavior changes [33].

Importantly, participants reported an increased meat-free self-efficacy, a marked shift toward reduced meat-eating and non–meat-eating identities, and a reduction in perception of meat consumption as the social norm. This reflects findings from a United Kingdom–based RCT that tested the effectiveness of a multicomponent behavioral intervention to reduce meat consumption [34]. That study found substantive reductions in meat intake (–61% at 4 weeks and –38% at 8 weeks in the intervention group) alongside increased intentions, positive attitudes, perceived control, and subjective norms of eating a low-meat diet. Previous research has suggested that meat-eating identity can explain intentions to reduce intake of red and processed meat [35], while higher levels of meat-free self-efficacy are an important predictor of successful meat-intake reduction [36]. We found no change in participants’ motivation to reduce meat intake, though this is likely because our participants had a high level of motivation at the start of the study, with those who reached week 5 having higher motivation than those who dropped out. Participants reported a decline in perceived social support during the program consistent with their reports of friends and family being one of the greatest barriers to performing their meat-intake reduction actions.

The results of our two OPTIMISE studies, taken together, suggest this online self-regulation program may be effective for helping motivated individuals to reduce their meat intake and closing the intention-behavior gap. In comparison to in-person interventions, there is preliminary evidence to support the scalability [37] and cost-effectiveness [38] of web-based interventions. Further, the OPTIMISE website uses a self-directed format (not requiring any researcher involvement), can be hosted at a minimal cost, and is currently publicly available and open to individuals who self-select to sign up. This approach is consistent with other web-based online programs designed to be made widely accessible at scale [39]. However, since its use is likely to be restricted to individuals with sufficient intrinsic motivation to seek out support for meat-intake reduction, this can only be one part of a wider strategy to support meat-intake reduction [40]. Population-level strategies that focus on restructuring the physical microenvironments (ie, choice architecture interventions or “nudges”) or economic environments to support a more plant-based diet are likely to be important complementary actions to support individual meat-intake reduction efforts [11,40].

### Conclusions

An online program to encourage self-monitoring of meat consumption, together with goal setting, educative feedback, action planning, and reflection may help individuals seeking to reduce their meat intake to change their diet and foster a reduced-meat or non–meat-eating identity. This type of support could be offered at scale with minimal cost and could complement other environmental interventions to help people eat less meat.

## Appendix

Multimedia Appendix 1 Health and environmental benefits presented to participants.

Multimedia Appendix 2 Meat consumption attitude questions.

Multimedia Appendix 3 Meat consumption reduction actions.

Multimedia Appendix 4 Example of weekly health and environmental feedback.

Multimedia Appendix 5 Participant characteristics of week 5 drop-outs vs completers.

Multimedia Appendix 6 Meat-eating identity proportions.

Multimedia Appendix 7 Predictors of change in total meat intake.

## Abbreviations

NDNS: National Diet and Nutrition Survey
OPTIMISE: Online Programme to Tackle Individual’s Meat Intake Through Self-regulation
RCT: Randomized Controlled Trial

## Data Availability

Data described in the manuscript will be made available upon request pending application and approval.
