# Supplementary material for: Evaluation of OPTIMISE (Online Programme to Tackle Individual’s Meat Intake Through Self-regulation): Cohort Study
Source: J Med Internet Res. 2022 Dec 12;24(12):e37389. doi: 10.2196/37389 (PMC9793298; doi:10.2196/37389)

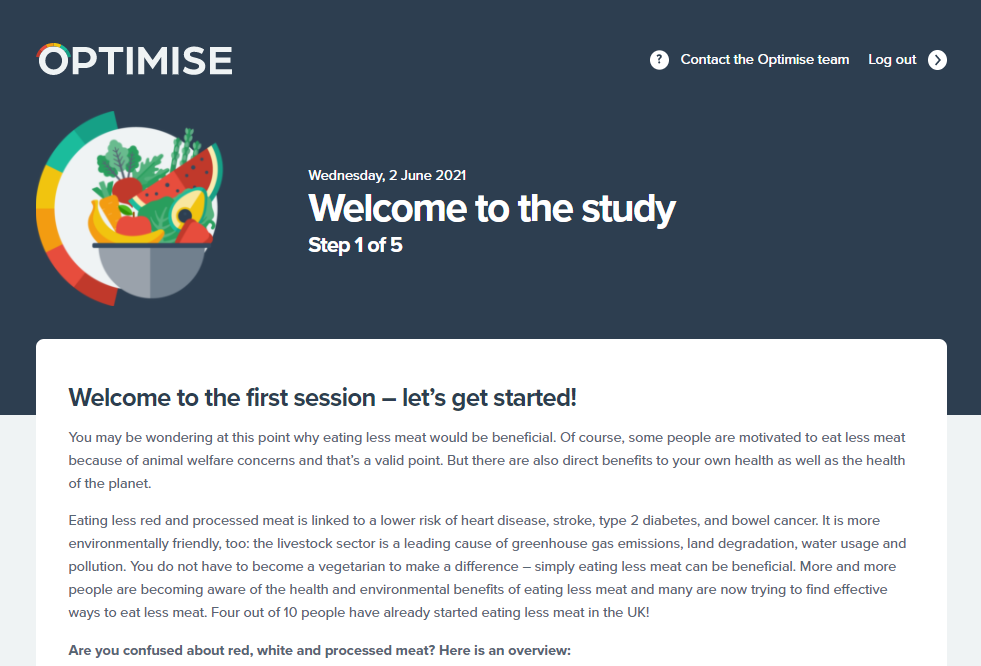

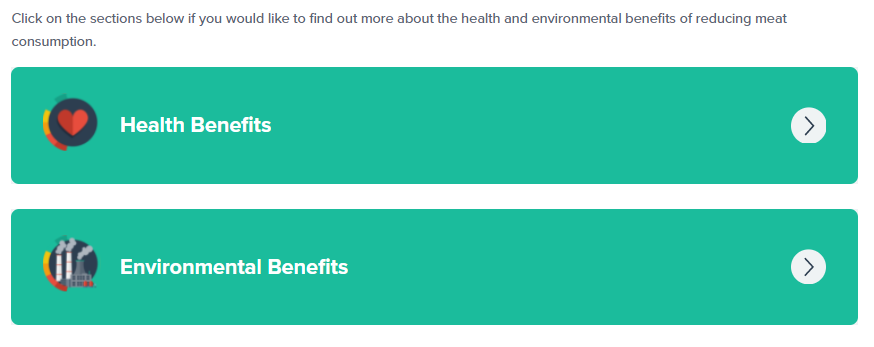
Health and environmental information presented to participants when they first registered with the OPTIMISE study website


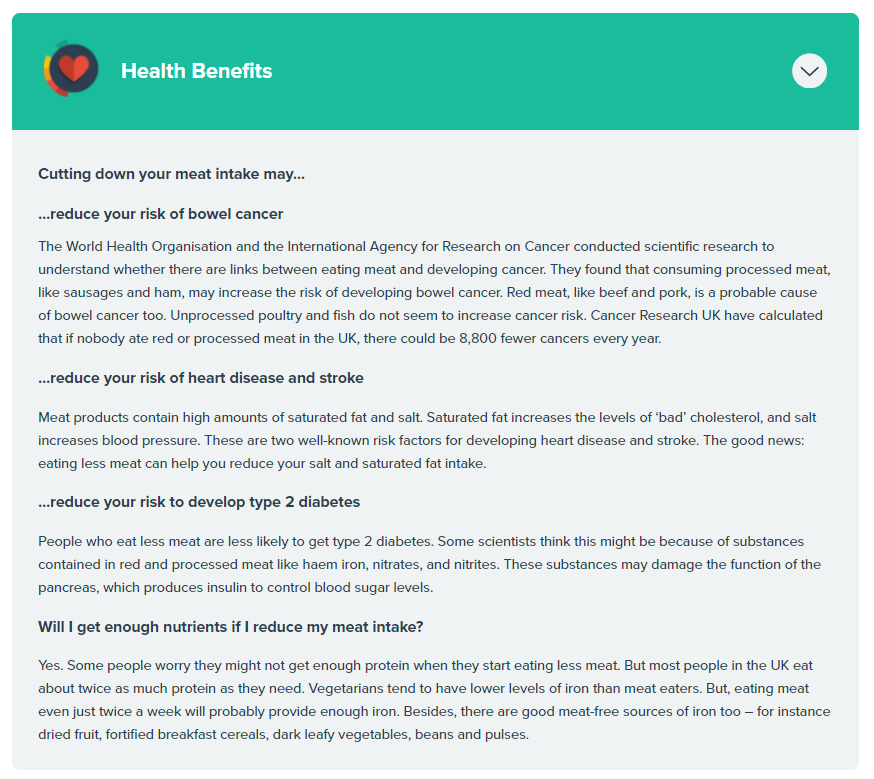


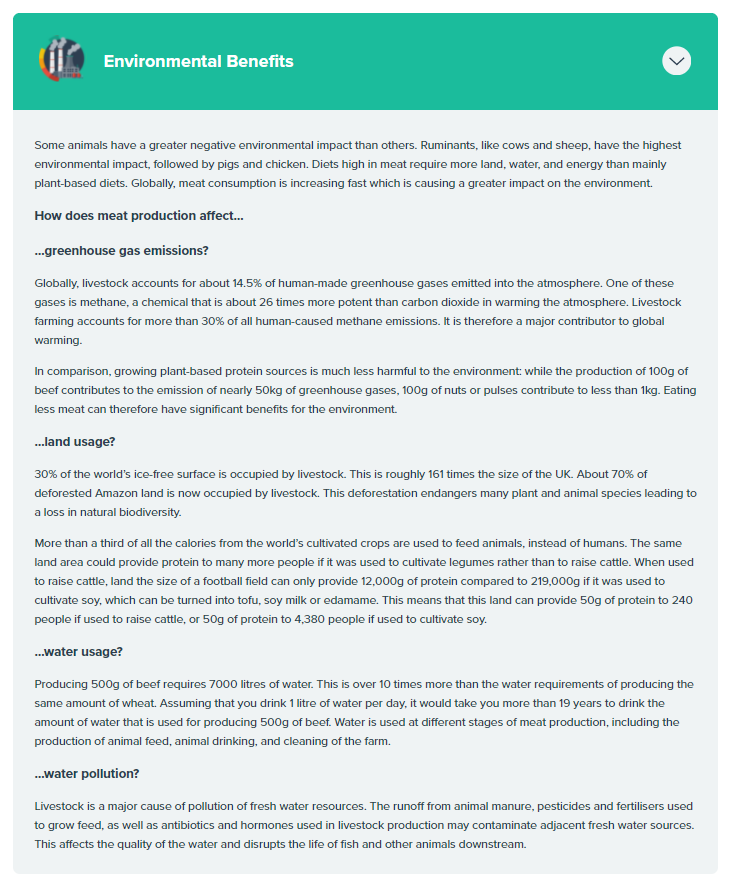

Supplement: Multimedia Appendix 1 [file jmir_v24i12e37389_app1.doc]
