# Supplementary material for: Evaluation of OPTIMISE (Online Programme to Tackle Individual’s Meat Intake Through Self-regulation): Cohort Study
Source: J Med Internet Res. 2022 Dec 12;24(12):e37389. doi: 10.2196/37389 (PMC9793298; doi:10.2196/37389)
Supplement: Multimedia Appendix 2 [file jmir_v24i12e37389_app2.docx]

Meat consumption attitude questions. Asked at baseline, first follow-up (Week 5) and second follow-up (Week 9)

**Meat-eating identity**

“When it comes to your eating identity, which of these do you identify with the most?”

1. Meat eater
2. Omnivore
3. Flexitarian
4. Pescetarian
5. Vegetarian
6. Vegan
7. Other: *Free text*

Meat-eating identities were then grouped into three categories: i) non-meat-eating identity (meat eater, omnivore); ii) reduced meat-eating identity (flexitarian); and iii) non-meat-eating identity (pescetarian, vegetarian and vegan). ‘Other’ entries were explored and manually assigned into one of these three categories.

**Meat-free self-efficacy**

“Please indicate on a scale from 1 “strongly disagree” to 7 “strongly agree” how much you agree with the following statements.”

1. I lack the cooking skills to prepare meat-free meals.
2. I don't know what to eat instead of meat.
3. I don't have enough willpower to not eat meat.

This scale was adapted from Lacroix & Gillford’s self-efficacy scale^1^. Mean meat-free self-efficacy scores were calculated at each time point.

**Meat reduction motivation**

“How motivated are you to reduce your meat intake beyond the context of this programme?”

- Scale from 1 (not at all motivated) – 10 (extremely motivated)

“How important, if at all, are each of the following factors in your consideration to reduce your overall meat intake?”

- Scale from 1 (not at all important) to 10 (extremely important)
- Health benefits
- Help the environment
- Animal welfare concerns
- High cost of meat
- Taste preferences
- Weight control
- Other: *Free text*

**Meat consumption social norms**

Based on the “4 Ns” – that meat consumption is *natural, necessary, normal* and *nice^2^*

Please indicate on a scale from 1 “strongly disagree” to 7 “strongly agree” how much you agree with the following statements.

1.       It is perfectly natural to eat meat. (Natural)

2.       A healthy diet requires at least some meat. (Necessary)

3.       Amongst people I know, it is normal to eat meat. (Normal)

4.       Meat adds so much flavour to a meal it does not make sense to leave it out. (Nice).

**Meat reduction social support**

How willing are the people you share your meals with to reduce their meat consumption?

- Scale from 1 (not open at all) – 10 (very open to it)

**References**

1. Lacroix K, Gifford R. Reducing meat consumption: Identifying group-specific inhibitors using latent profile analysis. Appetite. 2019;138:233-41. doi: 10.1016/j.appet.2019.04.002
2. Piazza J, Ruby MB, Loughnan S, Luong M, Kulik J, Watkins HM, et al. Rationalizing meat consumption. The 4Ns. Appetite. 2015;91:114-28. doi: 10.1016/j.appet.2015.04.011
