# Supplementary material for: Evaluation of OPTIMISE (Online Programme to Tackle Individual’s Meat Intake Through Self-regulation): Cohort Study
Source: J Med Internet Res. 2022 Dec 12;24(12):e37389. doi: 10.2196/37389 (PMC9793298; doi:10.2196/37389)

Example of weekly health and environmental feedback presented to participants provided during weeks 2-5.


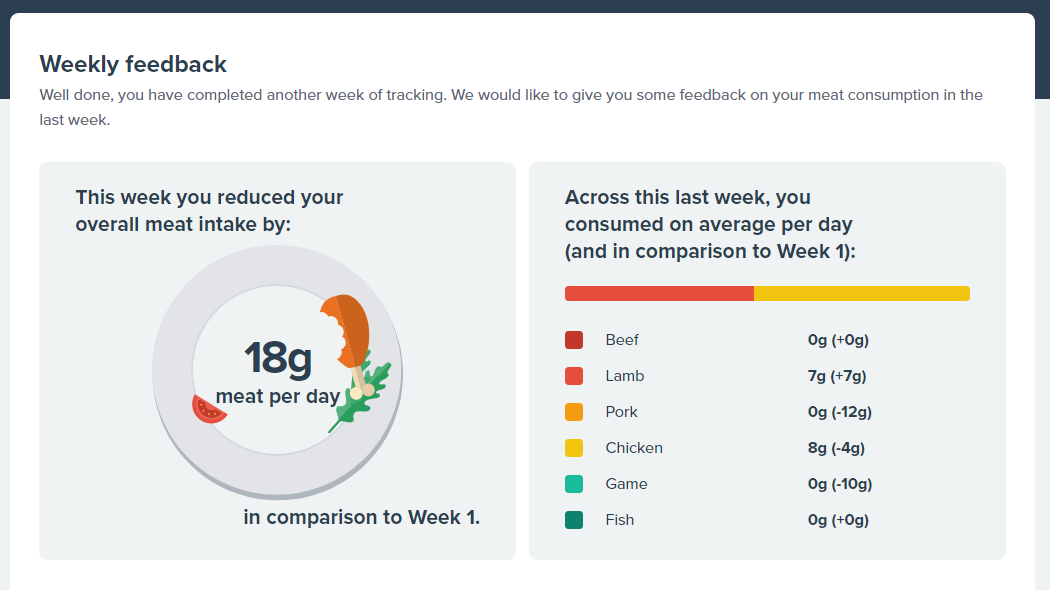


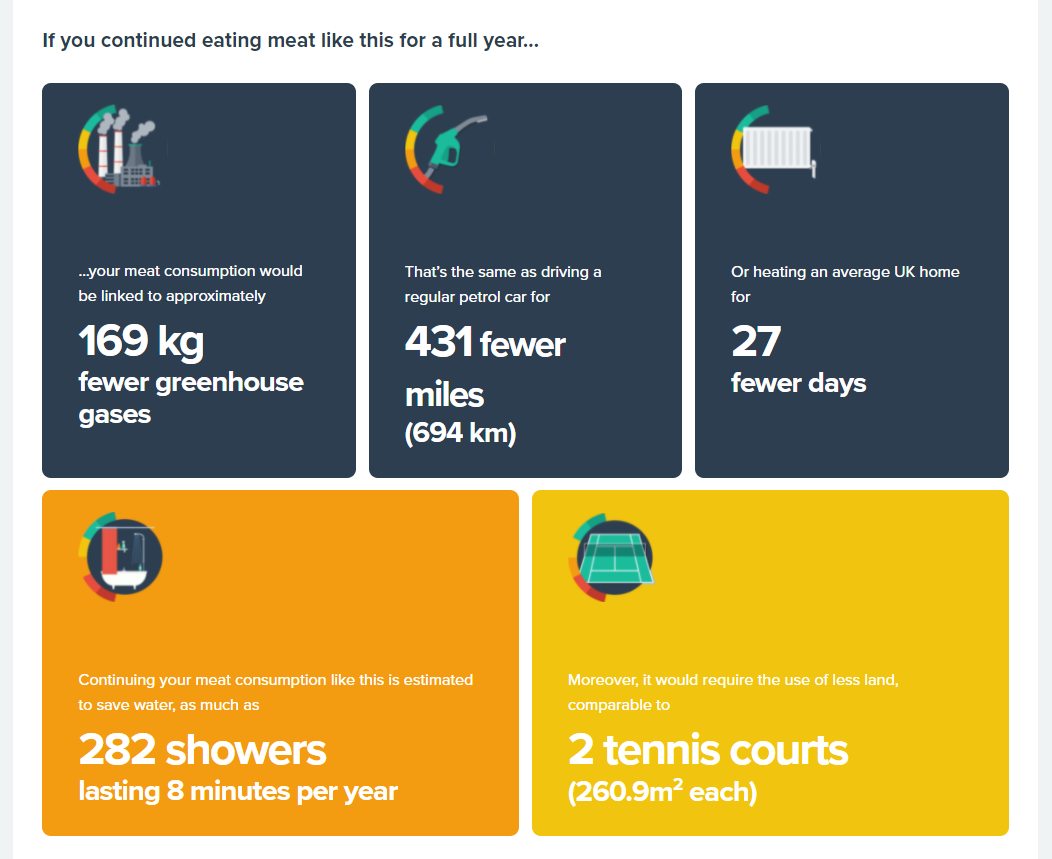


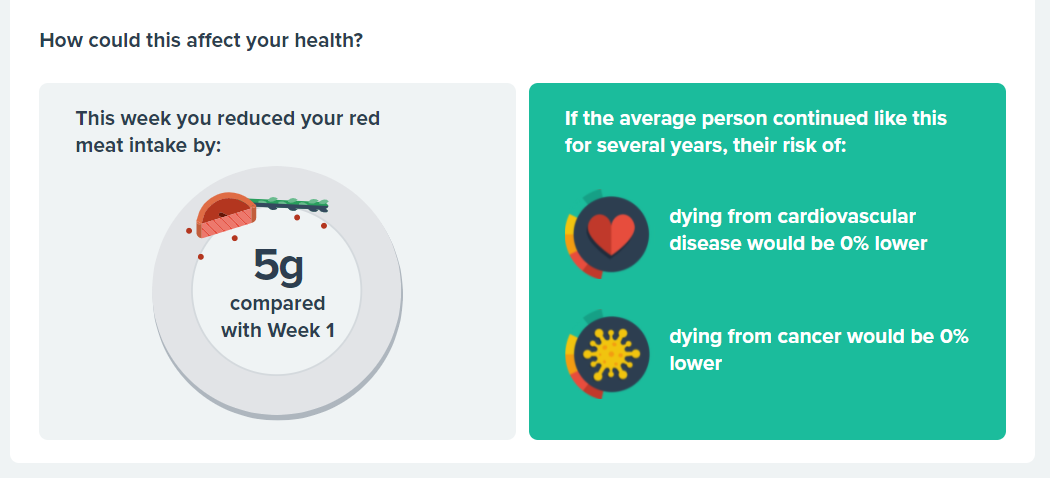

Supplement: Multimedia Appendix 4 [file jmir_v24i12e37389_app4.doc]
