# Supplementary material for: Evaluation of OPTIMISE (Online Programme to Tackle Individual’s Meat Intake Through Self-regulation): Cohort Study
Source: J Med Internet Res. 2022 Dec 12;24(12):e37389. doi: 10.2196/37389 (PMC9793298; doi:10.2196/37389)
Supplement: Multimedia Appendix 5 [file jmir_v24i12e37389_app5.docx]

Participant characteristics of the total baseline sample, and a comparison of week 5 (first follow-up) drop-outs and completers.

|  | Baseline Sample  (n=289) | Week 5  drop-outs (n=212) | Week 5  completers  (n=77) | Difference drop-outs vs completers (*P**) |
| --- | --- | --- | --- | --- |
| Age, mean (SD) | 46.8 (12.8)  (Min-Max: 18-84) | 47.3 (13.7)  (Min-Max: 18-84) | 45.5 (14.1)  (Min-Max: 21-78) | .322 |
| Gender, n (%) |  |  |  | .232 |
| Female | 209 (72) | 148 (70) | 61 (79) |  |
| Male | 78 (27) | 62 (29) | 16 (21) |  |
| Other/prefer not to say | 2 (0.1) | 2 (1) | 0 (0) |  |
| Ethnicity, n (%) |  |  |  | .811 |
| White-British | 165 (57) | 124 (58) | 41 (53) |  |
| White-Other | 84 (29) | 60 (28) | 24 (31) |  |
| Asian or Asian-British | 17 (6) | 13 (6) | 4 (5) |  |
| Black or Black-British | 4 (1) | 3 (1) | 1 (1) |  |
| Mixed/Other | 18 (6) | 11 (5) | 7 (9) |  |
| Prefer not to say | 1 (0.4) | 1 (0.5) | 0 (0) |  |
| Highest Educational Qualification, n (%) |  |  |  | .626 |
| University degree, NVQ level 4-5 or equivalent, and above | 242 (84) | 174 (82) | 68 (88) |  |
| Other post high school qualifications | 15 (5) | 10 (5) | 5 (6) |  |
| A’ levels, NVQ level 2-3 or equivalent | 21 (7) | 19 (9) | 2 (3) |  |
| Apprenticeship | 1 (0.4) | 1 (0.5) | 0 (0) |  |
| GCSE, NVQ level 1, or equivalent | 2 (0.1) | 2 (1) | 0 (0) |  |
| Other vocational, work-related qualifications | 3 (1) | 2 (1) | 1 (1) |  |
| No formal qualifications | 1 (0.4) | 1 (0.5) | 0 (0) |  |
| Prefer not to say | 4 (1) | 3 (1) | 1 (1) |  |
| Household size, n (%) |  |  |  | .911 |
| 1 | 55 (19) | 41 (19) | 14 (18) |  |
| 2 | 115 (40) | 81 (38) | 34 (44) |  |
| 3 | 57 (20) | 45 (21) | 12 (16) |  |
| 4 | 48 (17) | 35 (17) | 13 (17) |  |
| 5 | 10 (3) | 7 (3) | 3 (4) |  |
| 6+ | 4 (1) | 3 (1) | 1 (1) |  |
| Annual household income, n (%) |  |  |  | .064 |
| <£15,000 | 10 (3) | 6 (3) | 4 (5) |  |
| £15,000 - £24,999 | 24 (8) | 14 (7) | 10 (13) |  |
| £25,000 - £39,999 | 45 (16) | 31 (15) | 14 (18) |  |
| £40,000 - £75,000 | 99 (34) | 83 (39) | 16 (21) |  |
| >£75,000 | 90 (31) | 64 (30) | 26 (34) |  |
| Prefer not to say | 21 (7) | 14 (7) | 7 (9) |  |
| Currently trying to lose weight, n (%) |  |  |  | .026 |
| Yes | 198 (69) | 153 (72) | 45 (58) |  |
| No | 91 (31) | 59 (28) | 32 (42) |  |
| Dietary restrictions**, n (%) |  |  |  | .408 |
| Dairy-free | 14 (5) | 10 (5) | 4 (5) |  |
| Gluten-free | 19 (7) | 15 (7) | 4 (5) |  |
| Fish and shellfish allergy | 3 (1) | 2 (1) | 1 (1) |  |
| None | 259 (90) | 188 (89) | 71 (92) |  |
| Meat consumption (g/day), mean (SD) |  |  |  |  |
| Total meat | 146 (162) | 156 (185) | 118 (64) | .427 |
| Red meat | 53 (65) | 54 (71) | 49 (44) | .245 |
| Processed meat | 40 (80) | 44 (92) | 30 (28) | .629 |
| Red and processed meat | 92 (121) | 97 (136) | 79 (64) | .541 |
| Attitudes towards meat consumption, mean (SD) |  |  |  |  |
| Meat-free self-efficacy | 3.2 (1.2) | 3.2 (1.2) | 3.1 (1.2) | .305 |
| Meat reduction motivation | 7.5 (1.6) | 7.4 (1.5) | 7.8 (1.7) | .020 |
| Meat consumption social norm | 4.4 (1.0) | 4.4 (1.1) | 4.4 (0.9) | .742 |
| Meat reduction social support | 6.6 (2.5) | 6.5 (2.5) | 7.1 (2.5) | .056 |
| Meat-eating identity, n (%) |  |  |  | .346 |
| Meat-eater | 202 (70) | 149 (70) | 53 (69) |  |
| Meat-reducer | 82 (28) | 58 (27) | 24 (31) |  |
| Non-meat-eater | 5 (2) | 5 (2) | 0 (0) |  |

**P*-values estimated from two-tailed independent t-tests and Mann Whitney-U tests for normal and skewed continuous dependent variables, respectively, and chi-square tests for categorical dependent variables.

**Participants could select multiple answers.
