# Supplementary material for: Evaluation of OPTIMISE (Online Programme to Tackle Individual’s Meat Intake Through Self-regulation): Cohort Study
Source: J Med Internet Res. 2022 Dec 12;24(12):e37389. doi: 10.2196/37389 (PMC9793298; doi:10.2196/37389)
Supplement: Multimedia Appendix 6 [file jmir_v24i12e37389_app6.docx]

Meat-eating identity proportions at baseline and both follow-ups

| **Meat-eating identity** | **Expected percentage** | **Expected frequency** | **Observed frequency** | **Observed percentage** | **Chi-square** | ***P*-value** |
| --- | --- | --- | --- | --- | --- | --- |
| *First follow-up (Week 5; n=52)* |  |  |  |  | 10.68 | .005 |
| Meat-eater | 69 | 36 | 23 | 44 |  |  |
| Meat-reducer | 31 | 16 | 26 | 50 |  |  |
| Non-meat-eater | 0 | 0 | 3 | 6 |  |  |
| *Second follow-up (Week 9; n=40)* |  |  |  |  | 12.93 | .002 |
| Meat-eater | 73 | 29 | 17 | 43 |  |  |
| Meat-reducer | 27 | 11 | 20 | 50 |  |  |
| Non-meat-eater | 0 | 0 | 3 | 8 |  |  |

Estimates are from chi-square goodness of fit tests, comparing proportions at follow-ups with baseline (n=289). Sample size reflects the number of participants who answered the meat-eating identity question in the attitudes towards meat questionnaire, at each respective follow-up.
