# Supplementary material for: Evaluation of OPTIMISE (Online Programme to Tackle Individual’s Meat Intake Through Self-regulation): Cohort Study
Source: J Med Internet Res. 2022 Dec 12;24(12):e37389. doi: 10.2196/37389 (PMC9793298; doi:10.2196/37389)
Supplement: Multimedia Appendix 7 [file jmir_v24i12e37389_app7.docx]

Possible predictors of change in total meat intake from baseline to first follow-up (week 5)

|  | B | 95% CI | *P*-value |
| --- | --- | --- | --- |
| Age | -0.1 | -1.2, 0.9 | .844 |
| Gender, male | 33.9 | -0.4, 68.1 | .053 |
| Ethnicity |  |  |  |
| White-British | 37.4 | -20.5, 95.2 | .199 |
| White-Other | 53.6 | -6.2, 113.5 | .078 |
| Black or Black-British | 118.9 | -13.6, 251.3 | .077 |
| Mixed/Other | 40.2 | -28.6, 109.0 | .245 |
| Highest Educational Qualification |  |  |  |
| University degree, NVQ level 4-5 or equivalent, and above | 0.9 | -83.8, 85.5 | .984 |
| Other post high school qualifications | 1.2 | -95.1, 97.6 | .979 |
| Other vocational, work-related qualifications | 14.5 | -124.0, 152.9 | .834 |
| Prefer not to say | 37.8 | -97.7, 173.3 | .576 |
| Household size |  |  |  |
| 2 | 28.4 | -9.3, 66.1 | .136 |
| 3 | 4.6 | -45.4, 54.6 | .854 |
| 4 | 34.7 | -10.5, 79.8 | .129 |
| 5 | -42.8 | -121.6, 36.0 | .279 |
| 6+ | omitted |  |  |
| Annual household income |  |  |  |
| <£15,000 | -2.9 | -73.1, 67.4 | .934 |
| £25,000 - £39,999 | -9.7 | -59.8, 40.4 | .699 |
| £40,000 - £75,000 | 18.4 | -34.8, 71.7 | .488 |
| >£75,000 | 4.1 | -40.8, 49.0 | .854 |
| Prefer not to say | -7.1 | -71.3, 57.0 | .824 |
| Currently trying to lose weight, yes | 4.6 | -23.9, 33.0 | .748 |
| Dietary restrictions |  |  |  |
| Dairy-free | 1.3 | -103.5, 106.1 | .980 |
| Gluten-free, dairy-free | 19.5 | -50.8, 89.7 | .579 |
| Fish and shellfish allergy | -22.7 | -146.4, 101.1 | .713 |
| Gluten-free | -31.4 | -140.0, 77.2 | .563 |
| Meat consumption (g/day) | -0.9 | -1.1, -0.7 | <.001 |
| Attitudes towards meat consumption |  |  |  |
| Meat-free self-efficacy | -4.8 | -18.1, 8.6 | .474 |
| Meat reduction motivation | -6.9 | -16.1, 2.2 | .135 |
| Meat consumption social norm | 5.9 | -10.7, 22.5 | .476 |
| Meat reduction social support | -1.3 | -7.0, 5.3 | .691 |
| Meat-eating identity, meat-reducer | -16.6 | -51.7, 18.5 | .345 |
| Intervention engagement |  |  |  |
| Engagement tertiles, highest | -62.6 | -144.0, 18.7 | .128 |
| No. of action categories tried |  |  |  |
| 1 | 103.8 | 10.1, 197.5 | .031 |
| 2 | -0.1 | -75.3, 75.1 | .997 |
| 3 | 8.7 | -51.2, 68.6 | .770 |
| 4 | -12.3 | -61.8, 37.1 | .617 |
| 5 | -2.3 | -53.1, 48.6 | .928 |

Estimates from a multivariable linear regression model with change in meat intake as the dependent variable and all possible predictors included in one single model. Reference categories were: female, Asian/Asian British ethnicity, A’ levels, NVQ level 2-3 or equivalent, household size 1, annual household income £15,000 - £24,999, not trying to lose weight, no dietary restrictions, meat-eaters, middle engagement tertile, and six action categories tried.
